# Concordance between Thioacetamide-Induced Liver Injury in Rat and Human In Vitro Gene Expression Data

**DOI:** 10.3390/ijms21114017

**Published:** 2020-06-04

**Authors:** Patric Schyman, Richard L. Printz, Shanea K. Estes, Tracy P. O’Brien, Masakazu Shiota, Anders Wallqvist

**Affiliations:** 1 DoD Biotechnology High Performance Computing Software Applications Institute, Telemedicine and Advanced Technology Research Center, U.S. Army Medical Research and Development Command, Fort Detrick, MD 21702, USA; sven.a.wallqvist.civ@mail.mil; 2 The Henry M. Jackson Foundation for the Advancement of Military Medicine Inc. (HJF), Bethesda, MD 20817, USA; 3 Department of Molecular Physiology and Biophysics, Vanderbilt University School of Medicine, Nashville, TN 37232, USA; richard.printz@vanderbilt.edu (R.L.P.); shanea.estes@vanderbilt.edu (S.K.E.); tracy.obrien@vanderbilt.edu (T.P.O.)

**Keywords:** predictive toxicology, RNA-seq, thioacetamide, toxicogenomics, fibrosis, in vitro–in vivo correlations, interspecies correlation

## Abstract

The immense resources required and the ethical concerns for animal-based toxicological studies have driven the development of in vitro and in silico approaches. Recently, we validated our approach in which the expression of a set of genes is uniquely associated with an organ-injury phenotype (injury module), by using thioacetamide, a known liver toxicant. Here, we sought to explore whether RNA-seq data obtained from human cells (in vitro) treated with thioacetamide-S-oxide (a toxic intermediate metabolite) would correlate across species with the injury responses found in rat cells (in vitro) after exposure to this metabolite as well as in rats exposed to thioacetamide (in vivo). We treated two human cell types with thioacetamide-S-oxide (primary hepatocytes with 0 (vehicle), 0.125 (low dose), or 0.25 (high dose) mM, and renal tubular epithelial cells with 0 (vehicle), 0.25 (low dose), or 1.00 (high dose) mM) and collected RNA-seq data 9 or 24 h after treatment. We found that the liver-injury modules significantly altered in human hepatocytes 24 h after high-dose treatment involved cellular infiltration and bile duct proliferation, which are linked to fibrosis. For high-dose treatments, our modular approach predicted the rat in vivo and in vitro results from human in vitro RNA-seq data with Pearson correlation coefficients of 0.60 and 0.63, respectively, which was not observed for individual genes or KEGG pathways.

## 1. Introduction

The current gold standard for the evaluation of chemical toxicity is based on animal testing. Increasingly, however, high-throughput in vitro experiments and in silico approaches are becoming valuable complements to animal experiments for identifying the mechanisms underlying chemical-induced toxicity [1,2,3,4,5,6,7]. Two major questions in toxicology have yet to be satisfactorily resolved. First, does the cellular response induced by a toxic chemical (i.e., toxicant) in vitro correlate with the injury response induced by that toxicant in vivo? Second, how well does the toxicant-induced response in a non-human animal translate to that in a human? Interspecies comparisons are complex due to differences in genetics and bioavailability [8,9,10]. Many compounds may be toxic for one species but benign for others [11]. For example, theobromine, the compound that gives chocolate its bitter taste, can be lethal for dogs and cats, but not for humans, who have an enzyme that easily metabolizes it [12].

One way to try to bridge the gap between in vivo and in vitro results is to analyze the genomic response induced by a toxicant, i.e., toxicogenomics. In the field of toxicogenomics, a common assumption is that toxicity is associated with a change in the expression of either a single gene or a set of genes (i.e., a gene module) [13,14,15,16], and that chemical exposures leading to the same injury endpoint cause similar changes in gene expression. 

Using gene expression data from the Open Toxicogenomics Project-Genomics Assisted Toxicity Evaluation System (TG-GATEs) database, which contains data from Sprague–Dawley rats exposed to different chemicals for 4 to 29 days [17], we derived 11 and 8 chemical-induced injury modules (i.e., gene modules, each uniquely associated with a specific organ-injury phenotype) for the liver and kidney, respectively, based on the histopathological injury phenotypes documented in the same database [18]. In a subsequent study, we validated these injury modules in vivo by treating Sprague–Dawley rats with thioacetamide [19], an organosulfur compound extensively used in animal studies as a fibrosis-promoting liver toxicant [20,21,22,23]. Thioacetamide is highly toxic because it is rapidly metabolized in vivo by cytochrome P450 and flavin-containing monooxygenases into reactive metabolites (thioacetamide-S-oxide to produce thioacetamide-S,S-dioxide and reactive oxygen species) [24]. Our approach indicated cellular infiltration and fibrosis as the liver-injury endpoints most affected by thioacetamide [19].

Recently, we found that our modular approach could predict in vivo injury endpoints from in vitro RNA sequence (RNA-seq) data with a strong correlation (*R*^2^ > 0.6) [25]. This study compared in vivo rat data with in vitro cellular data 24 h after treatment with thioacetamide-S-oxide, an intermediate metabolite of thioacetamide required to induce toxicity in vitro [24]. The top ranked liver-injury modules identified by our in vitro studies using thioacetamide-S-oxide agreed with those identified in vivo using thioacetamide, indicating that in vitro cell injury was also associated with changes in the expression of fibrosis-related gene sets. These results suggest that, the predictions of our co-expressed gene module approach are more robust than those of gene signatures for specific pathologies, because they rely on groups of genes rather than individual genes. 

**Figure 1:**
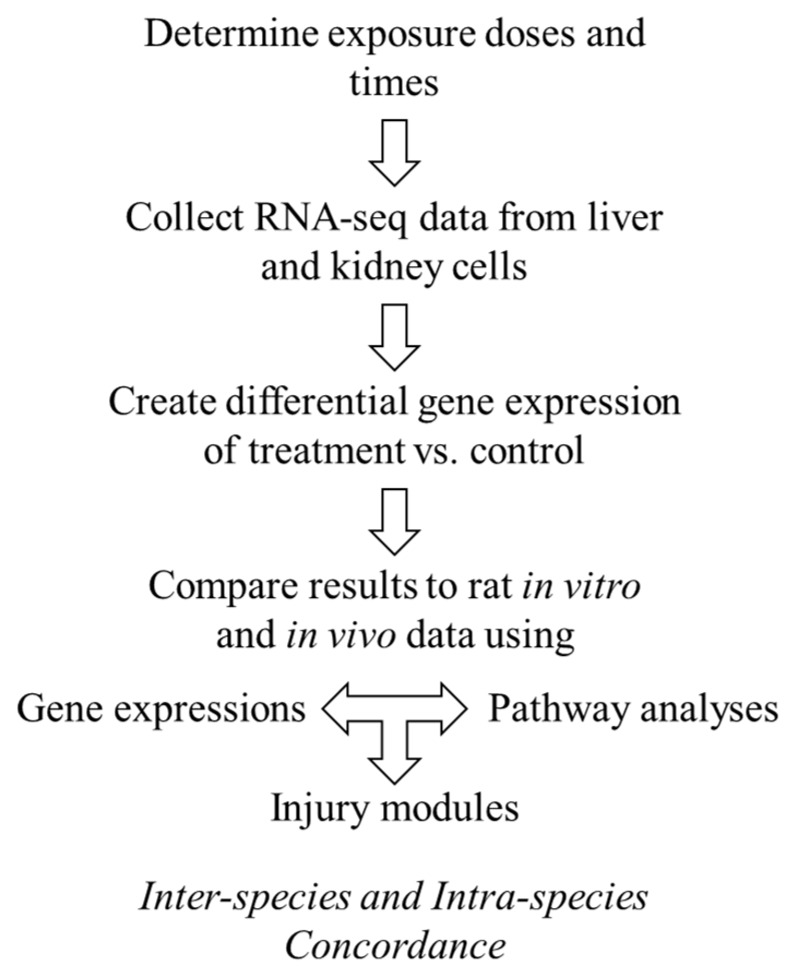
Diagram for comparing inter- and intra-species in vitro and in vivo cellular responses to low levels of thioacetamide exposures.

Here, we sought to explore the interspecies correlation using gene-expression responses in human cells exposed to thioacetamide-S-oxide in vitro with those induced by thioacetamide-S-oxide in cultured rat cells (in vitro) and by treating rats with thioacetamide (in vivo), following the diagram in Figure 1. We chose thioacetamide as a model compound since it is known to cause liver diseases (fibrosis, cirrhosis, and carcinoma) in humans and is frequently used to study fibrosis/cirrhosis in animals [26,27,28]. We used our injury modular approach to investigate interspecies correlations and compared it to gene-level and gene-pathway analyses. We found that the activation of our injury modules correlated well between humans and rats and between in vivo and in vitro studies, which was not the case at the individual gene or pathway level. To compare liver- and kidney-specific responses in vitro, we used two cell types from humans and rats—primary hepatocytes and renal tubule epithelial cells—and treated them with vehicle (control), a low dose of thioacetamide-S-oxide, or a high dose of the same compound.

## 2. Results

### 2.1. Analysis of Injury-Module Activation

We used the 8 kidney- and 11 liver-injury modules we identified and evaluated in previous work [18,19]. Several injury phenotypes may coexist, such as cellular infiltration and bile duct proliferation, which are early injury responses. However, for practical purposes, we grouped the injury modules into three general classes, inflammation, proliferation, and degeneration, and used the z-score values and a Fisher’s value of < 0.01 to identify the most likely injury.

#### 2.1.1. Activation of Liver-Injury Modules in Human Primary Hepatocytes

In human primary hepatocytes (Table 1), the fold-change (FC) based z-score values were calculated for each injury module after thioacetamide-S-oxide treatment, which resulted in significant activation of a few liver-injury modules (z-score values corresponding to a p-value < 0.05 and a Fisher’s value of < 0.01, indicated in bold). For each dose-time condition, the injury modules were grouped into inflammation, proliferation, and degeneration. Nine hours after low-dose thioacetamide-S-oxide treatment, only the nuclear alteration module was significantly activated (Table 1). Nine hours after high-dose treatment, the bile duct proliferation module was significantly activated, but other modules, such as cellular foci and fibrogenesis, tended to show some activation. Twenty-four hours after low-dose thioacetamide-S-oxide treatment, the single cell necrosis and cellular infiltration modules were significantly activated. Furthermore, 24 h after high-dose treatment, the cellular infiltration, bile duct proliferation, and nuclear alteration modules were significantly activated, indicating clear hepatic disturbances. Notably, 24 h after both low- and high-dose treatments, activation of the fibrogenesis module was already near the significance cutoff (*p*-value < 0.05). Liver fibrosis is a phenotype that develops over time, usually after repetitive damage to the liver.

#### 2.1.2. Activation of Kidney-Injury Modules in Human Renal Tubular Epithelial Cells

The gene expression response in human renal cells nine hours after either low- or high-dose thioacetamide-S-oxide treatment did not reveal any significantly activated kidney-injury modules (Table 2). The z-score value of the fibrogenesis module was significant, but the combined Fisher’s probability of 0.74 indicated considerable uncertainty in the FC values used for calculating module activation. The human renal cell response 24 h after low-dose thioacetamide-S-oxide treatment revealed an inflammatory response indicated by the activation of the cellular infiltration module, and high-dose treatment showed both an inflammatory and a degeneration response with the activation of the fibrogenesis and degeneration modules, respectively.

### 2.2. Correlation between Human and Rat Exposure Data

**Figure 2:**
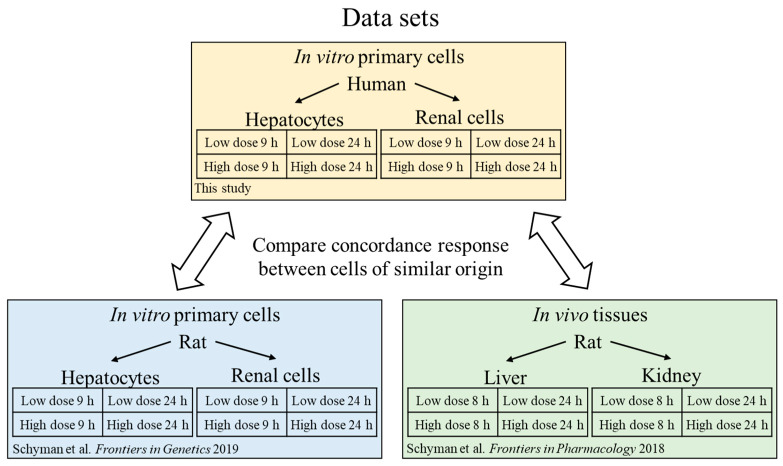
Data sets used in this study for comparing inter- and intra-species in vitro and in vivo cellular responses to low levels of thioacetamide exposures.

**Figure 3:**
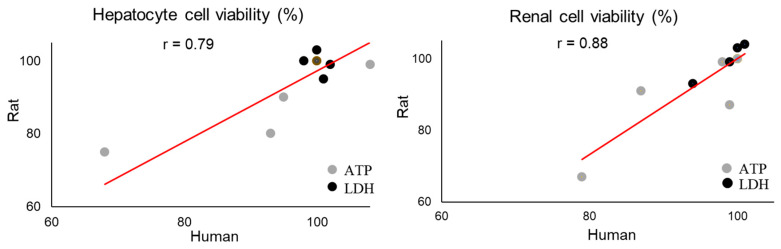
Pearson’s correlation (r) between rat and human adenosine triphosphate (ATP) and lactate dehydrogenase (LDH) in hepatocytes and renal cells after thioacetamide-S-oxide exposure *in vitro*.

To determine interspecies correlations, we compared the human in vitro results with results from our previous work on the effects of thioacetamide in cells from rats (in vivo) [19] and of thioacetamide-S-oxide in cultured rat cells (in vitro) [25]. Figure 2 presents an overview of the data collection we used for our analysis and all data are available in the supplementary files. To confirm that the doses used in rat and human in vitro are comparable we plotted the cell viability in Figure 3, all data are available in the Supplementary Material. The comparisons show dose dependent response for the adenosine triphosphate (ATP) and almost no change in the lactate dehydrogenase (LDH) measurements between human and rat. These results indicate that the doses are comparable between humans and rats in vitro and correspond to mild toxicity. The design of our rat in vivo study was to achieve minor liver injury after single dose of thioacetamide after 24 h, which is then comparable to our toxic exposures in vitro [19].

#### 2.2.1. Gene-Level Analysis of Human In Vitro, Rat In Vivo, and Rat In Vitro Data

Table 3 shows the number of differentially expressed genes (DEGs, as defined in the Materials and Methods Section) in human primary hepatocytes and renal tubular epithelial cells 9 or 24 h after low- or high-dose treatment with thioacetamide-S-oxide. For human in vitro exposures, the number of DEGs increased with dose and time, except at 24 h after high-dose treatment of renal cells. For comparative purposes, the bottom two rows of Table 3 also show the number of DEGs identified in vivo in the liver or kidney after exposure to a low- or high-dose thioacetamide treatment for 8 or 24 h in rats, and in vitro in primary rat hepatocytes and renal proximal tubular epithelial cells exposed to a low- or high-dose of thioacetamide-S-oxide for 9 or 24 h.

The numbers of DEGs in hepatocytes were similar between the human and rat in vitro data. In both humans and rats, the numbers of DEGs in renal cells were less than those in hepatocytes, which suggests that the kidney was less affected by thioacetamide/thioacetamide-S-oxide toxicity (Table 3). Some of the high numbers of DEGs in rat and human primary cells may be due to the higher dose used in the in vitro experiments as compared to the effective dose used in the rat in vivo studies [19,25].

#### 2.2.2. Module-Level Analysis of Human In Vitro, Rat In Vivo, and Rat In Vitro Exposure Studies

**Figure 4:**
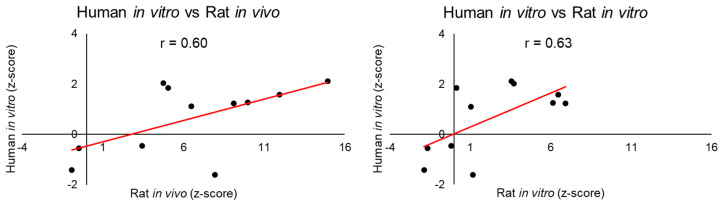
Pearson’s correlation (*r*) between liver-injury modules activated in rat and human hepatocytes after 24 h of high-dose treatment with thioacetamide-S-oxide (in vitro) or thioacetamide (in vivo).

Table 4 summarizes the correlations between the injury modules activated by exposing the liver or kidney to thioacetamide in rats or primary rat and human cells to thioacetamide-S-oxide, when comparing the human in vitro (human IVT) results with the rat in vitro (rat IVT) and in vivo (rat IVV) results (See Supplementary Material for all 24 graphs and tables). The highest correlations, 0.78 and 0.81, were between the in vitro and in vivo data (IVT-IVV) derived from rat cells and tissues collected 24 h after high- and low-dose exposure, respectively. Apart from two other exposure conditions, there was no significant positive correlation for any other dose-time cell-organ condition. The two exceptions involved activation of liver-injury modules 24 h after high-dose treatment, where Pearson’s correlation (*r*) between the liver-injury modules activated in human cells in vitro and those activated in either rat tissues in vivo or rat cells in vitro was at least 0.6 (Figure 4).

## 3. Discussion

Extensive efforts have been made to understand the frequently poor correspondence of experimental results between different species or between in vitro and in vivo studies [10,29,30,31,32]. We have previously used our toxicogenomic approach to predict rat in vivo toxicity endpoints from rat in vitro RNA-seq data with good concordance [25]. A persisting challenge, however, has been to understand the often weak interspecies correlations. Here we sought to address this using human and rat data. 

We examined the correlations between the injury modules activated in human-derived cells with those activated in rat tissues or cells. We found correlations between the liver-injury modules activated in human cells in vitro and those activated in the rat, both in vivo and in vitro, 24 h after high-dose treatment (Figure 4). This is in accord with the observation that, a chemical-induced injury requires time to develop into an injury-specific phenotype, such as liver fibrosis. In contrast, no significant positive interspecies correlations were found among the kidney-injury modules. These findings demonstrate the sensitivity of the modular approach, in that injury modules show low activation scores when no injury is observed. The interspecies correlation result is encouraging given the relatively weak toxicity response in human primary cells. Another limitation of using commercially available human primary cells is that they are frequently derived from a single donor with potentially preexisting conditions, and whose cause of death is often unknown.

**Figure 5:**
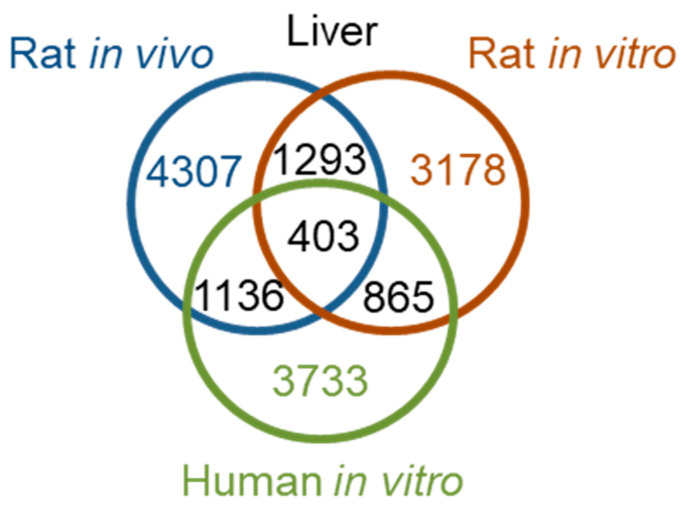
Overlap of differentially expressed genes (DEGs) 24 h after thioacetamide or thioacetamide-S-oxide treatment.

We examined the DEGs in the liver 24 h after high-dose treatment, where we observed interspecies correlations (Table 3). Notably, 24 h after thioacetamide/thioacetamide-S-oxide treatment, the numbers of DEGs in human primary hepatocytes, rat hepatocytes *in vivo*, and rat primary hepatocytes were comparable, amounting to 3733 (the number of rat genes orthologous to the 4267 human DEGs, identified using the *Ensemble* website [33]), 4307, and 3178, respectively. Figure 5 shows a Venn diagram of the interspecies overlap of DEGs. There were 403 DEGs common to all three datasets—about 10% of the total DEGs identified in human primary hepatocytes, rat liver in vivo, and rat primary hepatocytes. Although the pair-wise overlaps were roughly 2 to 3 times higher, many DEGs were not shared between species or between in vitro and in vivo studies. In fact, the numbers of overlapping genes were close to the numbers expected to overlap by chance and would result in poor interspecies correlation. To analyze the KEGG pathways in which the 403 overlapping DEGs are involved, we used the David Gene Functional Classification Tool [34,35]. Table 5 shows the 10 most enriched KEGG pathways. The most significantly enriched pathway was the complement and coagulation cascade pathway, which plays an important role in fibrosis. However, it is frequently involved in immune responses, and is therefore not injury specific. Therefore, any mechanistic interpretation across species based on KEGG pathways would be difficult. 

**Figure 6:**
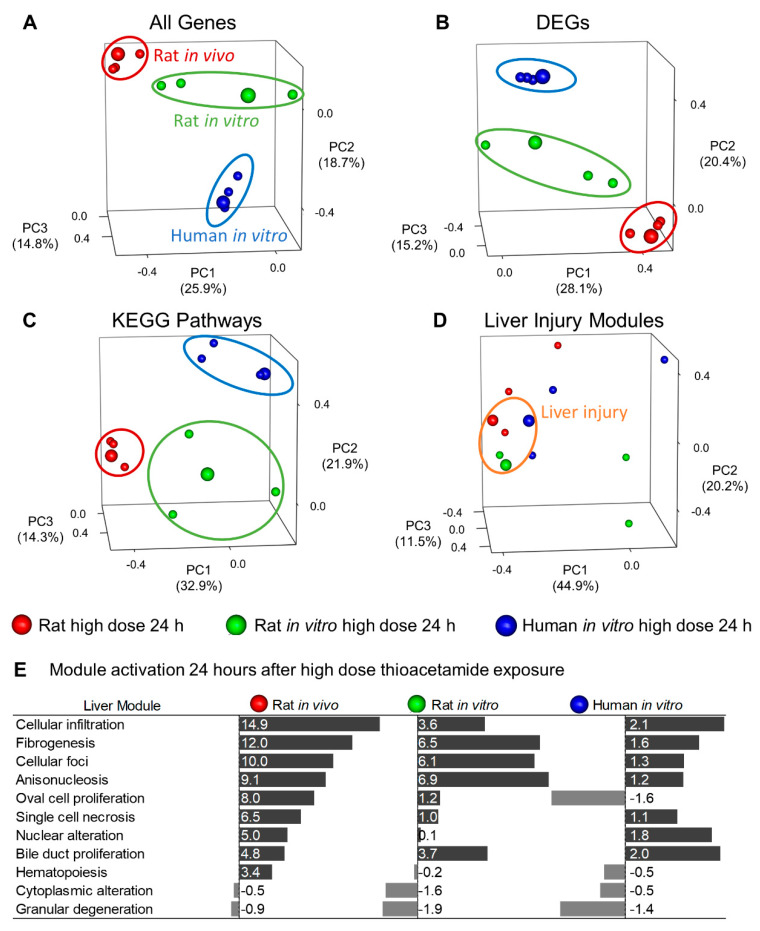
(**A**) Principal component analysis (PCA) using gene expression changes in liver samples obtained under four different conditions: low-dose 9-h (8 h for rat *in vivo*), high-dose 9-h (8 h for rat *in vivo*), low-dose 24-h, and high-dose 24-h treatment. The three enlarged spheres indicate the high-dose 24-h treatment for the three systems, and the ellipses highlight the clustering of conditions within each system. (**B**) PCA using 403 overlapping DEGs from Figure 2. (**C**) PCA using KEGG pathway activation for different conditions. (**D**) PCA using injury-module activation for different conditions. The orange ellipse highlights conditions where the modules indicate liver injury. (**E**) Graph showing relative liver-injury module activation for each system 24 h after high-dose treatment, ranked by the rat in vivo results.

We performed principal component analysis (PCA) to qualitatively compare commonly used approaches (e.g., DEG and KEGG pathway approaches) for analyzing in vitro, in vivo, and interspecies correlations. To facilitate interpretation of the PCA plots, we have enlarged the spheres corresponding to the high-dose 24-h condition, where we expect the highest toxicity. The PCA plots in Figure 6A–C show clustering of the rat in vivo, rat in vitro, and human in vitro data by systems rather than by conditions, as the three enlarged spheres were distinctly separated for each analysis approach.

Although we may have expected system-specific separation in the PCA plot for the individual-gene approach, we expected to see greater condition-specific separation in the plot for the KEGG pathway approach, which involves sets of genes. The KEGG Pathway plot illustrates that different KEGG pathways drive the principal components in different systems, making it difficult to mechanistically interpret an interspecies or in vitro–in vivo comparison. However, the injury module approach clustered the data points by conditions (enlarged spheres) associated with liver injury (orange ellipse). The small red and green spheres within the orange ellipse represent the low-dose 24-h conditions for the rat in vivo and in vitro data, respectively. The injury module approach properly clustered these two conditions with the large spheres, as our injury modules also predicted liver injury for both conditions. The PCA in Figure 6D illustrates that our modular approach to track injury was conserved across species and between in vitro and in vivo systems.

The primary liver-injury endpoint of thioacetamide is fibrosis. Although the fibrogenesis module was not the top-ranked injury module in the human in vitro assay (Figure 6E), other injury modules linked to known histopathological endpoints, such as those of bile duct proliferation and cellular infiltration, were activated [17]. Notably, 24 h after high-dose treatment in the human in vitro assay with thioacetamide-S-oxide, we observed bile duct proliferation and cellular infiltration (Figure 6E and Table 1), which are associated with fibrosis [36]. 

In our previous studies [19,25], we found that activation of the fibrogenesis module increased over time in the liver, an effect which we also observed here when comparing fibrogenesis module activation at 9 and 24 h in Table 1. The fibrogenesis module activation score would likely have continued to increase after 24 h, consistent with the observation that this phenotype takes longer than 24 h to become histologically visible. The delay in the fibrosis response in the human study relative to the fibrosis response in the rat studies may also reflect a species difference.

In human renal cells, 24 h of high-dose thioacetamide-S-oxide treatment activated the fibrogenesis and degeneration modules (Table 2). It is likely that these kidney-injury modules were activated within 24 h of thioacetamide-S-oxide exposure because a relatively high dose (1 mM) was required to detect clear indications of injury in human renal cells. In fact, the dose for the low-dose thioacetamide-S-oxide treatment of human renal cells (0.25 mM) was equivalent to that of the high-dose treatment of human primary hepatocytes, and the only low-dose activated kidney-injury module was cellular infiltration—a common response to chemical toxicity.

In summary, we show that using human in vitro cell responses based on RNA-seq data with an injury-module approach, rather than the commonly used DEGs/pathway approach, yields significant correlations with injury-module activation of rat in vivo and in vitro responses to a similar exposure of the toxicant, thioacetamide or its metabolite. Consequently, our injury-module approach could potentially be used to screen large numbers of chemicals in vitro and predict liver and kidney injuries, and thereby improve the efficiency of toxicity assessments by reducing the number of animals needed in experiments.

## 4. Materials and Methods 

### 4.1. Experimental Procedures

All experiments were conducted in accordance with the Guide for the Care and Use of Laboratory Animals of the United States Department of Agriculture, the Vanderbilt University Institutional Animal Care and Use Committee, and the U.S. Army Medical Research and Development Command Animal Care and Use Review Office. For the in vitro experiments, we purchased cryopreserved human hepatocytes from Triangle Research Labs (Research Triangle Park, NC, USA) and human renal proximal tubular epithelial cells from ScienCell Research Laboratories (Carlsbad, CA, USA). 

The preparation for the human hepatocyte experiment involved the following steps: 1) thaw and suspend the hepatocytes in thawing medium (MCHT50; Triangle Research Labs) at 6–7 million cells/50 mL; 2) centrifuge suspension at 50× *g* and resuspend cells in plating medium (MP250; Triangle Research Labs); 3) plate hepatocytes on collagen 1-coated 96-well plates at a density of 2 × 10^4^ cells/well for measurement of cell viability, and on collagen 1-coated 6-well plates at a density of 4.5 × 10^5^ cells/well for RNA-seq analysis; and 4) culture cells under 5% CO_2_ in an incubator at 37 °C for 4 h to allow cell attachment, and replace medium with hepatocyte maintenance medium (MM250, Triangle Research Labs). 

The preparation for the human renal proximal tubular epithelial cell experiment involved the following steps: 1) thaw and suspend renal proximal tubular epithelial cells in “Epithelial Cell Medium” (EpiCM, ScienCell Research Laboratories); 2) plate renal proximal tubular epithelial cells into poly-L-lysine-coated 96-well plates at a density of 2 × 10^4^ cells/well for measurement of cell viability, and on poly-L-lysine-coated 6-well plates at a density of 3 × 10^5^ cells/well for RNA collection; and 3) culture cells under 5% CO_2_ in an incubator at 37 °C for 4 h to allow cell attachment, and replace medium with the same medium.

We cultured both human hepatocytes and renal cells for an additional 18 h before adding thioacetamide-S-oxide or vehicle (maintenance medium; MM250 for hepatocytes and EpiCM for renal cells). For both cell types, we set the duration of exposure to vehicle or thioacetamide-S-oxide at 9 or 24 h. The low and high doses of thioacetamide-S-oxide were 0.125 and 0.25 mM, respectively, for hepatocytes, and 1.00 and 2.00 mM, respectively, for renal cells. We performed two cell viability assays in quintuplicates (*n* = 5) for each dose and time point. First, to measure cellular loss of lactate dehydrogenase (LDH), we collected cells and measured the cellular LDH activity remaining after each treatment using the Lactate Dehydrogenase Activity Assay Kit (Sigma-Aldrich, St. Louis, MO). Second, we measured cellular adenosine triphosphate (ATP) levels using the CellTier-Glo 2.0 Assay kit (Promega Co., Madison, WI, USA) according to the manufacturer’s protocol. Table 6 shows the relative viability of hepatocytes and renal proximal tubular epithelial cells at 9 or 24 h after exposure to thioacetamide compared to that of the corresponding vehicle-exposed cells. 

**Figure 7:**
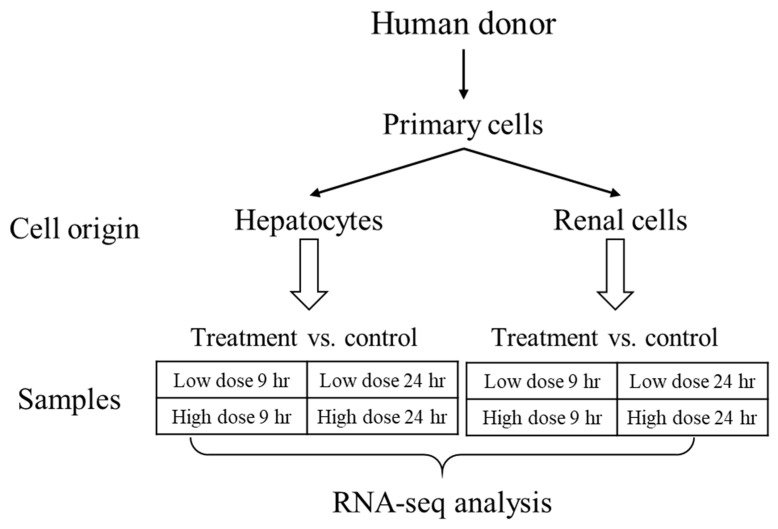
Data collection design for generating human in vitro gene expression signals from liver and kidney derived tissues.

We collected data according to Figure 7 and followed our previous procedure for isolating and sequencing RNA [19,25]. We first isolated total RNA from cultured cells using TRIzol Reagent (Thermo Fisher Scientific, Waltham, MA, USA) and the Direct-zol RNA MiniPrep kit (Zymo Research, Irvine, CA, USA). We then submitted the isolated RNA samples to the Vanderbilt University Medical Center VANTAGE Core (Nashville, TN, USA), which performed RNA quality determination and sequencing according to the following protocol: 1) assess total RNA quality using a 2100 Bioanalyzer (Agilent, Santa Clara, CA, USA); 2) use at least 200 ng of DNase-treated total RNA with high integrity to generate poly-A-enriched mRNA libraries, using KAPA Stranded mRNA sample kits with indexed adaptors (New England BioLabs, Beverly, MA, USA); 3) assess library quality using the 2100 Bioanalyzer (Agilent) and quantitate libraries using KAPA library Quantification kits (KAPA Biosystems); 4) subject pooled libraries to 150-bp double-end sequencing with the Illumina NovaSeq600 system (Illumina, San Diego, CA, USA) according to the manufacturer’s protocol; and 5) use the Bcl2fastq2 Conversion Software v2.20 (Illumina) to generate de-multiplexed Fastq files.

### 4.2. Analysis of RNA-Seq Data

We used the RNA-seq data analysis tool Kallisto for read alignment and quantification [37]. Kallisto pseudo-aligns the reads to a reference, producing a list of transcripts that are compatible with each read while avoiding alignment of individual bases. In this study, we pseudo-aligned the reads to the human transcriptome (GRCh38.p12) downloaded from the Ensemble website (http://www.ensembl.org/index.html) [33]. Kallisto achieves a level of accuracy similar to that of other competing methods, but is orders of magnitude faster. Its speed allows for the use of a bootstrapping technique to calculate uncertainties of transcript abundance estimates by repeating the analyses after resampling with replacement. In this study, we employed this technique to repeat the analysis 100 times. The files from RNA-seq analysis were deposited in NCBI’s Gene Expression Omnibus (GEO) database under series accession numbers GSE134641.

To identify differentially expressed genes (DEGs) from transcript abundance data, we used Kallisto’s companion analysis tool Sleuth, which uses the results of the bootstrap analysis during transcript quantification to directly estimate the technical gene variance for each sample [38]. We defined DEGs by using a false discovery rate adjusted *p*-value (*q*-value) of no more than 0.05 and a minimum gene expression *β*-value of 0.41 as the criteria for differential expression, which corresponds to a fold-change (FC) value of 1.5. Note that the *β*-value is defined as the natural logarithm of the effect size, and that the effect size and FC value of a gene are not equivalent. Nonetheless, the ranking and the directionality of change in gene expression (i.e., whether a gene is up- or down-regulated) should be the same. In the Supplemental Material, we provide the q-values of all genes and DEGs.

### 4.3. Module Activation Score

To identify gene sets that significantly change between treatment and control cohorts, we previously developed the aggregate absolute FC (AAFC) method to calculate the activation score for a gene set [19]. This method first calculates the FC value for each gene, i.e., the difference between the mean log-transformed gene-expression values for samples in the treatment and control cohorts. Subsequently, it calculates the absolute value of the log-transformed FC value for each gene, and calculates the total FC value of the absolute values for each gene set (e.g., module or pathway). 

Using the AAFC method, here we assessed the significance of the FC value for each gene set by Student’s t-test (*n* = 5 for both treatment and control cohorts) and calculated a combined *p*-value for each gene set (module) using Fisher’s method as an indicator of the robustness of the reliability of the genes in the module [39]. We then used the module scores to perform null hypothesis tests and estimate the significance of each module by its *p*-value, defined as the probability that the score for randomly selected FC values (10,000 times) is greater than the score from the actual module. A small *p*-value (< 0.05) implies that the module value is significant. The z-score is the number of standard deviations by which the actual module value differs from the mean of the randomly selected FC values (10,000 times). The z-score indicates the degree of module activation (i.e., the module activation score) and can be used to rank the modules.

## Figures and Tables

**Table 1:** Activation of liver-injury modules in human primary hepatocytes (in vitro) 9 and 24 h after exposure to thioacetamide-S-oxide (low: 0.125 mM; high: 0.25 mM).

	9 h				24 h			
Liver Injury Module	Low dose		High dose		Low dose		High dose	
**Inflammation**	z-score	Fisher’s	z-score	Fisher’s	z-score	Fisher’s	z-score	Fisher’s
Cellular infiltration	−0.3	6.9 × 10^−3^	1.4	2.1 × 10^−14^	**2.1 ^a^**	**2.3 × 10^−20^**	**2.1**	**5.0 × 10^−21^**
Fibrogenesis	−0.4	4.5 × 10^−5^	1.5	2.6 × 10^−23^	1.6	6.6 × 10^−29^	1.6	3.0 × 10^−28^
Hematopoiesis	−0.1	4.6 × 10^−2^	1.5	6.7 × 10^−21^	−2.2	9.8 × 10^−13^	−0.5	4.6 × 10^−15^
Single cell necrosis	−0.3	2.2 × 10^−3^	0.1	1.5 × 10^−14^	**3.4**	**1.1 × 10^−23^**	1.1	5.4 × 10^−14^
**Proliferation**								
Bile duct proliferation	−0.3	1.9 × 10^−2^	**2.1**	**1.1 × 10^−12^**	0.7	7.0 × 10^−14^	**2.0**	**7.6 × 10^−20^**
Cellular foci	−0.3	8.5 × 10^−2^	1.6	5.0 × 10^−12^	1.0	4.4 × 10^−13^	1.3	4.4 × 10^−17^
Oval cell proliferation	−0.5	4.4 × 10^−10^	−0.1	3.0 × 10^−81^	−1.8	1.9 × 10^−92^	−1.6	1.6 × 10^−103^
**Degeneration**								
Anisonucleosis	−0.6	2.7 × 10^−5^	0.2	5.4 × 10^−61^	−1.2	6.4 × 10^−62^	1.2	5.8 × 10^−76^
Cytoplasmic alteration	−0.1	9.6 × 10^−5^	0.1	4.0 × 10^−16^	−0.1	5.1 × 10^−16^	−0.5	3.8 × 10^−14^
Granular degeneration	−0.1	6.7 × 10^−2^	−2.9	2.2 × 10^−4^	−1.2	1.7 × 10^−12^	−1.4	3.0 × 10^−17^
Nuclear alteration	**2.1**	**4.5 × 10^−13^**	−0.5	9.0 × 10^−98^	1.0	6.5 × 10^−119^	**1.8**	**1.0 × 10^−138^**

^a^ Quantities in **bold** indicate significant activation of module (i.e., *p*-value < 0.05 and a Fisher’s value of < 0.01).

**Table 2:** Activation of kidney-injury modules in human renal tubular epithelial cells (*in vitro*) 9 and 24 h after exposure to thioacetamide-S-oxide (low: 0.25 mM; high: 1.0 mM).

	9 h				24 h			
Kidney Injury Module	Low dose		High dose		Low dose		High dose	
**Inflammation**	z-score	Fisher’s	z-score	Fisher’s	z-score	Fisher’s	z-score	Fisher’s
Cellular infiltration	−1.0	9.9 × 10^−1^	0.6	1.1 × 10^−14^	**2.2** ^a^	**3.1 × 10^−43^**	1.3	3.5 × 10^−29^
Fibrogenesis	2.8	7.4 × 10^−1^	0.2	1.9 × 10^−46^	1.6	7.1 × 10^−91^	**2.3**	1.4 × 10^−71^
Intracytoplasmic inclusion body	0.6	1.0 × 10^+0^	−0.5	2.8 × 10^−6^	1.4	6.8 × 10^−23^	1.4	2.3 × 10^−25^
								
**Proliferation**								
Hypertrophy	−2.0	9.3 × 10^−1^	1.1	1.3 × 10^−16^	−0.6	1.9 × 10^−9^	0.9	1.3 × 10^−23^
**Degeneration**								
Degeneration	−0.6	1.0 × 10^+0^	−1.6	5.8 × 10^−15^	1.6	3.3 × 10^−65^	**1.9**	**1.6 × 10^−49^**
Dilatation	0.1	1.1 × 10^−1^	−0.3	1.5 × 10^−6^	0.6	4.6 × 10^−8^	−1.1	5.6 × 10^−4^
Hyaline cast	−0.1	8.2 × 10^−1^	−0.8	1.9 × 10^−7^	1.3	4.7 × 10^−19^	−0.6	3.1 × 10^−9^
Necrosis	−0.5	1.0 × 10^+0^	0.5	6.2 × 10^−9^	1.2	1.6 × 10^−19^	1.4	5.5 × 10^−19^

^a^ Quantities in **bold** indicate significant activation of module (i.e., *p*-value < 0.05 and a Fisher’s value of < 0.01).

**Table 3:** Differentially expressed genes obtained from human primary hepatocytes and renal tubular epithelial cells after exposure to thioacetamide-S-oxide. The rat in vivo and in vitro data are from [25].

	Liver				Kidney			
	Low dose		High dose		Low dose		High dose	
	9 h	24 h	9 h	24 h	9 h	24 h	9 h	24 h
Human—in vitro	469	2421	2104	4267	40	1661	1789	1022
Rat—in vitro	259	4292	2159	3178	890	71	2575	3529
Rat—in vivo	3027 ^a^	1999	4443 ^a^	4307	257 ^a^	746	172 ^a^	1571

^a^ Rat in vivo data were collected after 8 h of thioacetamide treatment.

**Table 4:** Pearson correlation coefficients between injury modules activated by exposure to thioacetamide or its metabolite in human in vitro studies and those activated in rat in vitro and in vivo studies.

	Liver			Kidney		
	Rat IVT vs IVV ^a^	Human IVT vs Rat IVV	Human IVT vs Rat IVT	Rat IVT vs IVV	Human IVT vs Rat IVV	Human IVT vs Rat IVT
High dose 24 h	**0.78 ^b^**	**0.60**	**0.63**	−0.28	0.29	0.01
Low dose 24 h	**0.81**	0.16	0.34	−0.54	−0.72	0.03
High dose 8 h	−0.06	0.47	−0.12	0.17	0.39	0.19
Low dose 8 h	−0.66	0.12	−0.60	−0.61	0.41	−0.32

^a^ IVT: in vitro; IVV: in vivo. ^b^ Values in bold indicate significant positive correlations (*p*-value ≤ 0.05).

**Table 5:** KEGG pathways enriched in overlapping DEGs.

KEGG Pathway	Benjamini *p*-value
Complement and coagulation cascades	1.7 × 10^−8^
Metabolic pathways	1.9 × 10^−7^
Biosynthesis of antibiotics	2.6 × 10^−4^
Biosynthesis of amino acids	0.001
Carbon metabolism	0.001
Chemical carcinogenesis	0.002
Steroid hormone biosynthesis	0.003
Retinol metabolism	0.019
Selenocompound metabolism	0.022
PPAR signaling pathway	0.047

**Table 6:** Relative LDH and ATP levels to assess viability of human hepatocytes and renal proximal tubular epithelial cells exposed to thioacetamide-S-oxide for 9 or 24 h compared to vehicle-exposed cells at the same time points. Data are presented as mean ± standard error of the mean (SEM) (*n* = 5 per group).

		9 h of Exposure		24 h of Exposure	
		ATP	LDH	ATP	LDH
Type of cell	Dose (mM)	%	%	%	%
Hepatocytes	0 (vehicle)	100 ± 5	100 ± 8	100 ± 6	100 ± 4
	0.125	100 ± 13	100 ± 10	93 ± 15	98 ± 5
	0.25	95 ± 1	102 ± 10	68 ± 1	101 ± 5
Real Epithelial Cells	0 (vehicle)	100 ± 5	100 ± 8	100 ± 6	100 ± 5
	1.00	98 ± 8	100 ± 7	87 ± 4	99 ± 7
	2.00	91 ± 11	103 ± 15	79 ± 3	94 ± 5

## Supplementary Materials

Supplementary materials can be found at https://www.mdpi.com/1422-0067/21/11/4017/s1. The files generated from RNA-seq analysis have been deposited in NCBI’s Gene Expression Omnibus GEO database under series accession numbers GSE120195 (rat in vivo), GSE134569 (rat in vitro), and GSE134641 (human in vitro).

## Abbreviations

DEG	Differentially Expressed Gene
FC	Fold Change
AAFC	Aggregate Absolute Fold Change
ATP	Adenosine Triphosphate
LDH	Lactate Dehydrogenase
SEM	Standard Error of the Mean
PCA	Principal Component Analysis
IVT	In Vitro
IVV	In Vivo
